# Deep learning model for classifying endometrial lesions

**DOI:** 10.1186/s12967-020-02660-x

**Published:** 2021-01-06

**Authors:** YunZheng Zhang, ZiHao Wang, Jin Zhang, CuiCui Wang, YuShan Wang, Hao Chen, LuHe Shan, JiaNing Huo, JiaHui Gu, Xiaoxin Ma

**Affiliations:** Department of Obstetrics and Gynecology, Shengjing Hospital of China Medical University, 39 Huaxiang Road, Shenyang, 110021 People’s Republic of China

**Keywords:** Computer-aided diagnosis, Convolutional neural network, Endometrial lesion, Hysteroscopy, VGGNet

## Abstract

**Background:**

Hysteroscopy is a commonly used technique for diagnosing endometrial lesions. It is essential to develop an objective model to aid clinicians in lesion diagnosis, as each type of lesion has a distinct treatment, and judgments of hysteroscopists are relatively subjective. This study constructs a convolutional neural network model that can automatically classify endometrial lesions using hysteroscopic images as input.

**Methods:**

All histopathologically confirmed endometrial lesion images were obtained from the Shengjing Hospital of China Medical University, including endometrial hyperplasia without atypia, atypical hyperplasia, endometrial cancer, endometrial polyps, and submucous myomas. The study included 1851 images from 454 patients. After the images were preprocessed (histogram equalization, addition of noise, rotations, and flips), a training set of 6478 images was input into a tuned VGGNet-16 model; 250 images were used as the test set to evaluate the model’s performance. Thereafter, we compared the model’s results with the diagnosis of gynecologists.

**Results:**

The overall accuracy of the VGGNet-16 model in classifying endometrial lesions is 80.8%. Its sensitivity to endometrial hyperplasia without atypia, atypical hyperplasia, endometrial cancer, endometrial polyp, and submucous myoma is 84.0%, 68.0%, 78.0%, 94.0%, and 80.0%, respectively; for these diagnoses, the model’s specificity is 92.5%, 95.5%, 96.5%, 95.0%, and 96.5%, respectively. When classifying lesions as benign or as premalignant/malignant, the VGGNet-16 model’s accuracy, sensitivity, and specificity are 90.8%, 83.0%, and 96.0%, respectively. The diagnostic performance of the VGGNet-16 model is slightly better than that of the three gynecologists in both classification tasks. With the aid of the model, the overall accuracy of the diagnosis of endometrial lesions by gynecologists can be improved.

**Conclusions:**

The VGGNet-16 model performs well in classifying endometrial lesions from hysteroscopic images and can provide objective diagnostic evidence for hysteroscopists.

## Background

At the clinic, patients are often diagnosed with suspected endometrial lesion due to symptoms such as abnormal uterine bleeding or infertility [1, 2]. Transvaginal ultrasound and diagnostic hysteroscopy are common gynecological examinations to diagnose endometrial lesions conclusively [3–5]. Transvaginal ultrasound is usually the first choice, but it has low diagnostic specificity and does not enable physicians to obtain pathological tissue specimen; in some cases, further hysteroscopy is required. [3–5]. Diagnostic hysteroscopy is a minimally invasive examination through which the hysteroscopist can directly observe the endometrial lesions and normal endometrium in the patient’s uterine cavity, so that the gynecologist can make a more accurate primary diagnosis [6]. These endometrial lesions include endometrial polyps, submucous myomas, intrauterine adhesions, endometrial hyperplasia, malignancies, intrauterine foreign bodies, placental remnants, and endometritis [6]. An accurate primary diagnosis helps gynecologists to explain the condition to patients and decide on a primary treatment. However, the diagnostic performance of hysteroscopy for these lesions depends on the experience of the hysteroscopist, resulting in a degree of subjectivity in the gynecologist’s diagnosis [7]. A stable and objective computer-aided diagnosis (CAD) system could shorten the learning curve of inexperienced gynecologists and effectively reduce the subjectivity (interobserver error) of gynecologist diagnosis.

Deep learning is a discipline that has recently played a prominent role in fields such as computer vision, speech recognition, and natural language processing [8]. Many practices in the medical field have also benefited from the use of deep learning, including identifying potential depression patients in social networks and locating the cecum in surgical videos [9, 10]. Convolutional neural networks (CNNs) are a class of algorithms that excel in image classification tasks in deep learning, especially for classifying or detecting objects that can be directly observed [11]. It has been reported that CNNs can diagnose skin cancer at a level no less than that of experts [12]. The ability of CNNs to classify laryngoscopic images in most cases exceeds that of physicians [13]. There have been many other reports of endoscopic CAD systems based on deep learning, and excellent results have been achieved in cystoscopy, gastroscopy, enteroscopy, and colposcopy [14–17]. Deep learning has previously been applied in the field of hysteroscopy: Török reported the use of fully convolutional neural networks (FCNNs) to segment uterine myomas and normal uterine myometrium [18], and Burai used FCNNs to identify the uterine wall [19].However, no CNN-based CAD system for hysteroscopy has yet been reported.

This study considers the five most common endometrial lesions: endometrial hyperplasia without atypia (EH), including simple and complex hyperplasia; atypical hyperplasia (AH); endometrial cancer (EC); endometrial polyps (EPs); and submucous myomas (SMs) [20]. This study aimed to construct a CNN-based CAD system that can classify endometrial lesion images obtained from hysteroscopy and to evaluate the diagnostic performance of this model. The results show that the CAD system slightly outperforms gynecologists in classifying endometrial lesion images. It provides evidence of the feasibility of using artificial intelligence to assist in clinical diagnosis of endometrial lesions.

## Methods

### Dataset

This study retrospectively collected images of patients who underwent hysteroscopic examination at the Shengjing Hospital of China Medical University from 2017 to 2019, which confirmed the presence of endometrial lesions. All images were taken using an Olympus OTV-S7 (Olympus, Tokyo, Japan) endoscopic camera system with a resolution of 720 × 576 pixels and were stored in JPEG format. Images meeting the following criteria were excluded: (a) poor quality or unclear images; (b) images with no lesions in the field of view; (c) images with a large amount of bleeding in the field of view; (d) images from patients with an intrauterine device or who were receiving hormone therapy; (e) images from patients with multiple uterine diseases; and (f) images from patients without histopathological results. The resulting dataset included 1851 images from 454 patients, including 509 EH, 222 AH, 280 EC, 615 EP, and 225 SM images. We randomly extracted 250 images (50 images for each category) from the dataset as the testing and validation set, and the remaining images were used as the original training set for data augmentation and model training. Table 1 shows the detailed dataset partition used in this study. Subsequently, the test set was randomly divided into two parts (125 images per part) to explore the role of the model in assisting gynecologists to diagnose endometrial lesions. This study was approved by the Ethics Committee of Shengjing Hospital (No. 2017PS292K).

**Table 1:** Partition details of the endometrial lesion dataset for classification

Category	Dataset		Training set		Test set
	No. of patients	No. of images	No. of original images	No. of augmented images	No. of test images
EH	124	509	459	915	50
AH	53	222	172	1032	50
EC	66	280	230	1055	50
EP	148	615	565	825	50
SM	63	225	175	1050	50
Total	454	1851	1601	4877	250

*AH* atypical hyperplasia, *EC* endometrial cancer, *EH* endometrial hyperplasia without atypia, *EP* endometrial polyp, *SM* submucous myoma

### Data preprocessing

**Fig. 1:**
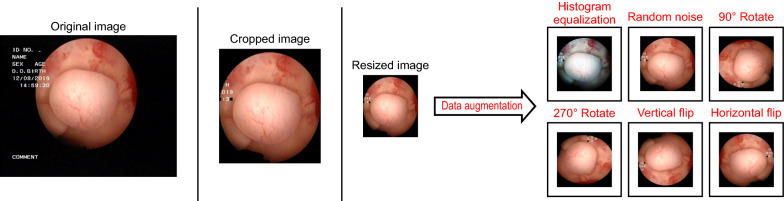
Example of image preprocessing. We first cropped and resized all images in the dataset. Then, we augmented the resized training set to increase the amount of data, allowing us to improve the model’s generalization ability and robustness

All images were manually cropped by gynecologists to remove excessive non-lesion regions and retain the region of interest, thus preventing irrelevant features from disturbing the performance of the deep learning model. To improve the generalizability and robustness of the deep learning model, we performed data augmentation on the training set, including color histogram equalization, random addition of salt-and-pepper noise, 90° and 270° rotations, and vertical and horizontal flips (Fig. 1). The final training set was augmented from 1601 to 6478 images. The test set was not processed. Finally, all images were resized to 224 × 224 pixels and rescaled for training, validating, and testing.

### Convolutional neural network and transfer learning

We selected VGGNet [21] as the main structure of our deep learning model and tuned it to implement transfer learning [22]. VGGNet was developed by the Oxford Visual Geometry Group and won second place in the image classification task of the 2014 ImageNet Large Scale Visual Recognition Challenge (ILSVRC) [23]. It has a top-5 accuracy of 92.3% in classifying 1000 object categories. Compared to AlexNet [24], the winner of ILSVRC 2012, VGGNet uses a smaller convolution kernel and deepens the network to achieve better results [23]. VGGNet-16 and VGGNet-19 are commonly used versions of VGGNet. There is no significant difference in the effect of the two in application, but VGGNet-16 has fewer layers and parameters than VGGNet-19 [21]. This provides VGGNet-16 with shorter processing time and lower storage space usage than VGGNet-19, so we selected VGGNet-16 as our model network.

**Fig. 2:**
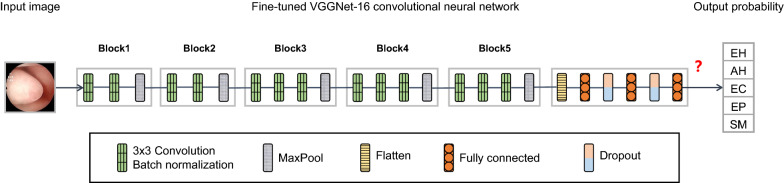
Structure of the fine-tuned VGGNet-16 model. Our network structure is a tuned VGGNet-16 model. The data stream flows from left to right, and the cross-entropy loss is calculated from the prediction results of each category and their corresponding probabilities. The model iterates repeatedly to reduce the loss value, thereby improving its accuracy

We employed the VGGNet-16 CNN, pretrained on ImageNet, and adjusted its 4096 neurons in the fully connected layer to 512 neurons and its 1000-category output layer to 5 categories. We added a batch normalization layer after each convolutional layer to improve the training speed of the model [25]. Important training parameters were set as follows: (a) the input shape was 224 pixel × 224 pixel × 3 channel; (b) the batch size was 64; (c) the number of training epochs was 200; and (d) the optimizer used was stochastic gradient descent (SGD) with a learning rate of 0.00001 and a momentum of 0.9. The structure of our CNN is shown in Fig. 2, and a summary of the model is shown in Additional file 1: Table S1. The CNN for this research was built using the open source Keras neural network library [26]. Our fine-tuned VGGNet-16 CNN was used for transfer learning and endometrial lesion classification task.

### Performance evaluation metrics

To evaluate the diagnostic performance of the CNN model, one chief physician with more than 20 years of experience and two attending physicians with more than 10 years of experience in hysteroscopic examination and surgery diagnosed lesions using all images in the test set without knowing the histopathological results. These diagnoses were compared with the diagnostic results of the CNN model.

To explore the auxiliary role of the model in the diagnosis of endometrial lesions by gynecologists, three other licensed gynecologists performed direct diagnosis and model-aided diagnosis on two randomly divided test sets without knowing the histopathological results.

We present the results in two ways: five-category and two-category classification. In the first task, each lesion was classified as EH, AH, EC, EP, or SM. In the second task, lesions were categorized as premalignant/malignant (AH and EC) or benign (EH, EP, and SM).

The diagnostic performance of the model and that of gynecologists is initially demonstrated using confusion matrix, which records the samples in the test set according to their true and predicted categories in the form of a matrix, but it is not a direct evaluation metric. The actual evaluation metrics used in this study were derived from the confusion matrix, which shows the numbers of true positive (TP), false positive (FP), false negative (FN), and true negative (TN) classifications. The secondary evaluation metrics calculated from the primary evaluation metrics are as follows:

$$ {\text{Sensitivity}}\left( {{\text{TPR}}} \right) = {\text{TP}}/\left( {{\text{TP}} + {\text{FN}}} \right); $$

$$ {\text{Specificity}}\left( {{\text{TNR}}} \right) = {\text{TN}}/\left( {{\text{TN}} + {\text{FP}}} \right); $$

$$ {\text{Precision}}\left( {\text{P}} \right) = {\text{TP}}/\left( {{\text{TP}} + {\text{FP}}} \right); $$

$$ {\text{F1 - Score}} = {2} \times {\text{P}} \times {\text{TPR}}/\left( {{\text{P}} + {\text{TPR}}} \right); $$

$$ {\text{Accuracy}} = {5} \times \Sigma {\text{TPi}}/\Sigma \left( {{\text{TPi}} + {\text{FPi}} + {\text{FNi}} + {\text{TNi}}} \right); $$

$$ \text{Area under the curve (AUC): the area under the receiver operating characteristic (ROC) curve.}$$

All calculation and visualization operations were implemented in Python Version 3.7.0.

## Results

**Fig. 3:**
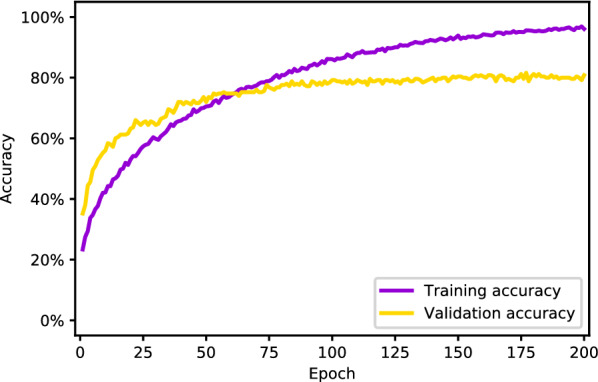
Training and validation accuracy by training epochs of VGGNet-16 convolutional neural network. During training, the overall accuracy of the model on the training and validation sets increases as the model iterates. The model’s performance plateaus on the training and validation sets at epochs 190 and 90, respectively

During training, the model’s accuracy changed with increase in epochs, as shown in Fig. 3. After 90 epochs, the validation accuracy plateaued.

### Five-category classification task

**Fig. 4:**
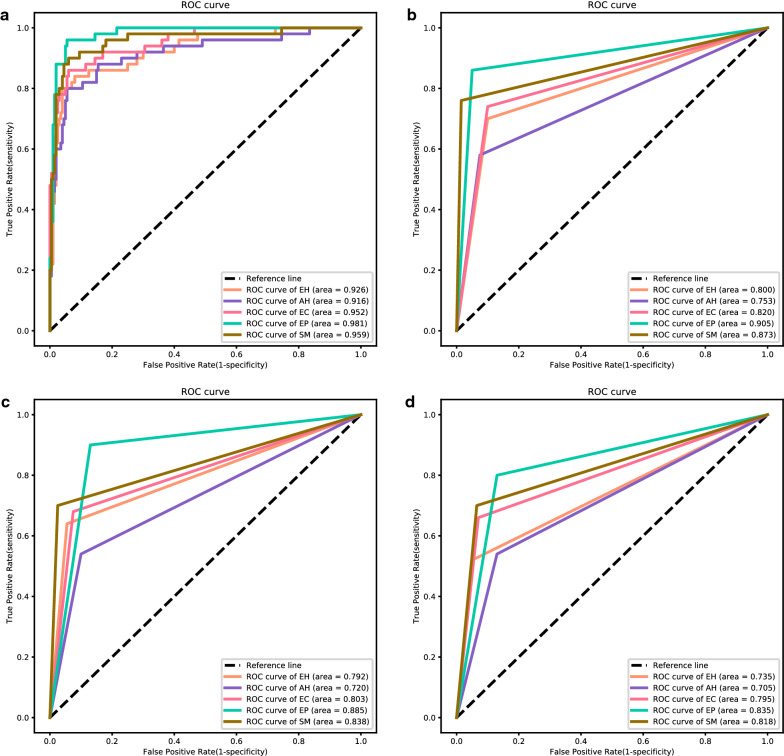
Five-category ROC curves of the VGGNet-16 model and gynecologists. Five-category receiver operating characteristic (ROC) curves: **a**, **b**, **c**, and **d** are the ROC curves of VGGNet-16 and gynecologists 1, 2, and 3, respectively. *AH* atypical hyperplasia, *EC* endometrial cancer, *EH* endometrial hyperplasia without atypia, *EP* endometrial polyp, *SM* submucous myoma

**Fig. 5:**
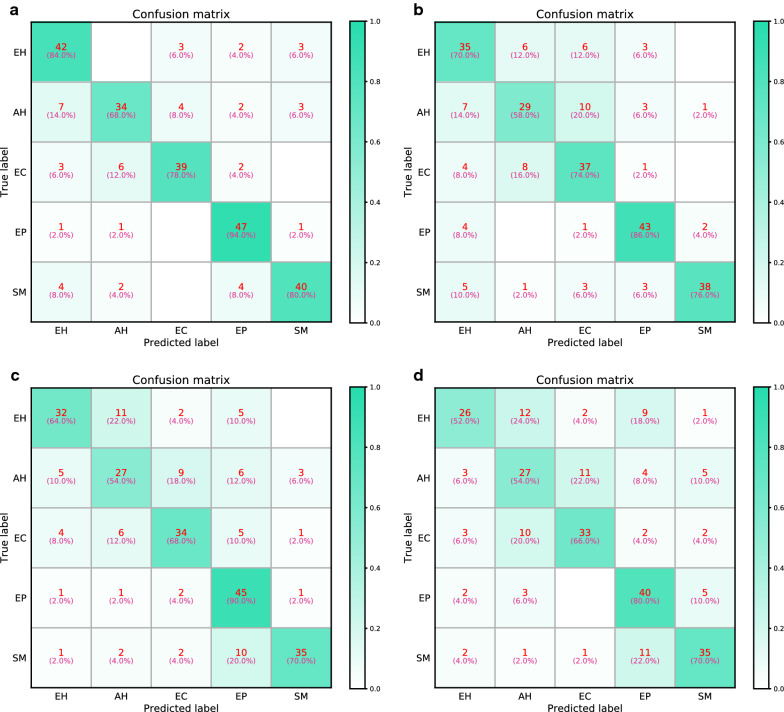
Confusion matrices of the VGGNet-16 model and gynecologists. Confusion matrices: **a**, **b**, **c**, and **d** are the confusion matrices of the VGGNet-16 model and gynecologists 1, 2, and 3 in classifying the test set, respectively. The x axes are the predicted labels, which are the diagnoses made by the model or gynecologists. The y axes are the true labels, which is the histopathological result. The number in each small square represents the corresponding number of images with the same predicted true label and its percentage of the total number of images under the true label. *AH* atypical hyperplasia, *EC* endometrial cancer, *EH* endometrial hyperplasia without atypia, *EP* endometrial polyp, *SM* submucous myoma

For the five-category classification task, the VGGNet-16 model achieves an accuracy of 80.8%. The model’s sensitivity and specificity for diagnosing EH lesions are 84.0% and 92.5% (AUC = 0.926), 68.0% and 95.5% (AUC = 0.916) for AH lesions, 78.0% and 96.5% for EC (AUC = 0.952), 94.0% and 95.0% for EP (AUC = 0.981), and 80.0% and 96.5% for SM (AUC = 0.959). The accuracies of the three gynecologists were 72.8%, 69.2%, and 64.4%. Detailed five-category diagnostic performance evaluation metrics are shown in Table 2. The five-category ROC curves of the model and gynecologists are shown in Fig. 4. The confusion matrices of the VGGNet-16 model and three gynecologists are shown in Fig. 5. The VGGNet-16 model slightly outperforms the three gynecologists in accurately diagnosing endometrial lesions.

**Table 2:** Diagnostic performance of the VGGNet-16 model and gynecologists in the five-category classification task

Category	Sensitivity (%)	Specificity (%)	Precision (%)	F1-score (%)	AUC	Accuracy (%)
VGGNet-16						
EH	84.0	92.5	73.7	78.5	0.926	80.8
AH	68.0	95.5	79.1	73.1	0.916	
EC	78.0	96.5	84.8	81.3	0.952	
EP	94.0	95.0	82.5	87.9	0.981	
SM	80.0	96.5	85.1	82.5	0.959	
Gynecologist 1						
EH	70.0	90.0	63.6	66.7	0.800	72.8
AH	58.0	92.5	65.9	61.7	0.753	
EC	74.0	90.0	64.9	69.2	0.820	
EP	86.0	95.0	81.1	83.5	0.905	
SM	76.0	98.5	92.7	83.5	0.873	
Gynecologist 2						
EH	64.0	94.5	74.4	68.8	0.792	69.2
AH	54.0	90.0	57.4	55.7	0.720	
EC	68.0	92.5	69.4	68.7	0.803	
EP	90.0	87.0	63.4	74.4	0.885	
SM	70.0	97.5	87.5	77.8	0.838	
Gynecologist 3						
EH	52.0	95.0	72.2	60.5	0.735	64.4
AH	54.0	87.0	50.9	52.4	0.705	
EC	66.0	93.0	70.2	68.0	0.795	
EP	80.0	87.0	60.6	69.0	0.835	
SM	70.0	93.5	72.9	71.4	0.818	

*AH* atypical hyperplasia, *AUC* area under the receiver operating characteristic (ROC) curve, *EC* endometrial cancer, *EH* endometrial hyperplasia without atypia, *EP* endometrial polyp, *SM* submucous myoma

**Fig. 6:**
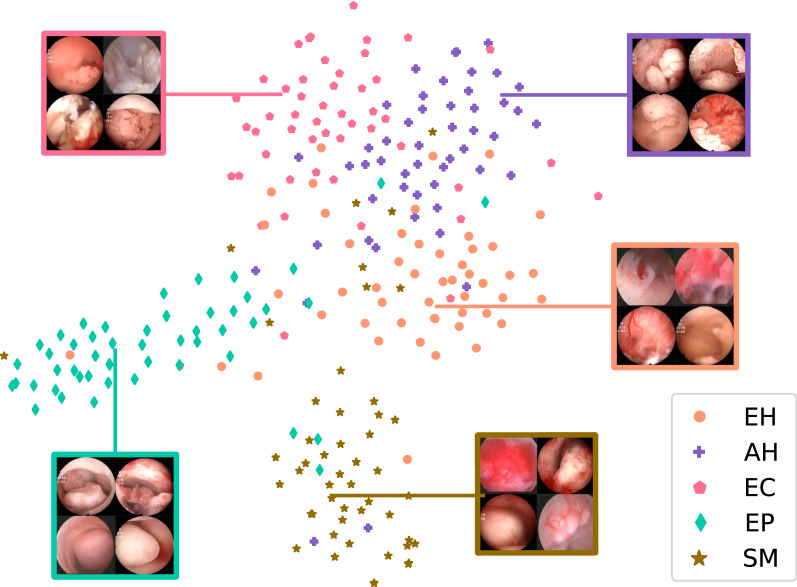
Dimension-reduced scatter plot of the last fully connected layer of the VGGNet-16 model. We output the 512-dimensional data of all images in the test set at the last fully connected layer of the optimal model and applied the t-SNE algorithm to reduce the data to two dimensions and show them in a scatter plot, along with some example images. *AH* atypical hyperplasia, *EC* endometrial cancer, *EH* endometrial hyperplasia without atypia, *EP* endometrial polyps, *SM* submucous myoma

**Fig. 7:**
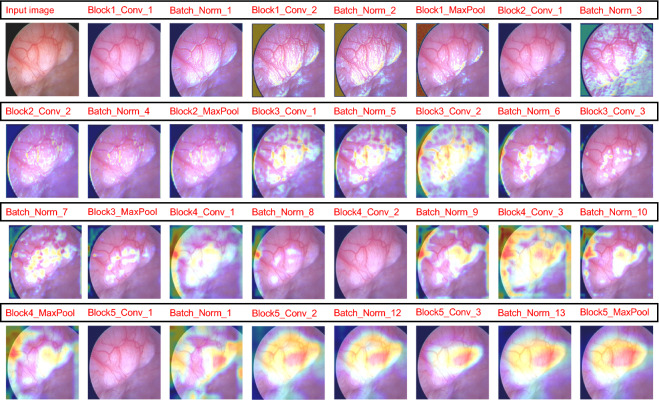
Feature heatmaps of a submucous myoma image output by the VGGNet-16 model. The sum feature maps output by each convolutional layer, batch normalization layer, and MaxPool layer of the VGGNet-16 model for a submucous myoma image in the test set were up-sampled and superimposed on the original image and displayed as feature heatmaps

**Fig. 8:**
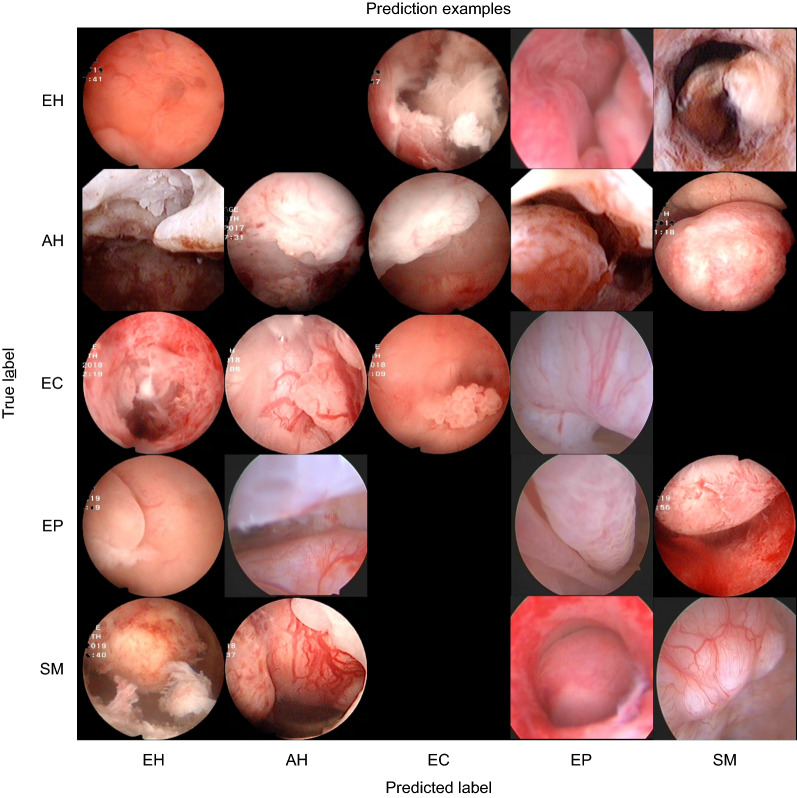
Example classification results output by the VGGNet-16 model. The x axis is the predicted label of the model’s output and the y axis is the histopathology result of these images. *AH* atypical hyperplasia, *EC* endometrial cancer, *EH* endometrial hyperplasia without atypia, *EP* endometrial polyp, *SM* submucous myoma

To directly observe the clustering of the five types of lesions, we applied the t-distributed stochastic neighbor embedding (t-SNE) [27] dimension reduction algorithm. The 512-dimensional output of all images in the test set of the last fully connected layer was reduced to two dimensions and is displayed in Fig. 6. We can see from this figure that most of the images are mapped on their own fixed areas, but there is an area of overlap between EH, AH, and EC. To deepen our understanding of the CNN’s calculation process, we output the sum feature maps of an SM image in the test set at each convolutional layer, batch normalization layer, and MaxPool layer of the VGGNet-16 model and superimposed them on the original image after upsampling these sum feature maps. The superimposed heatmaps are shown in Fig. 7. Some examples of the model’s classification are shown in Fig. 8.

### Two-category classification task

**Fig. 9:**
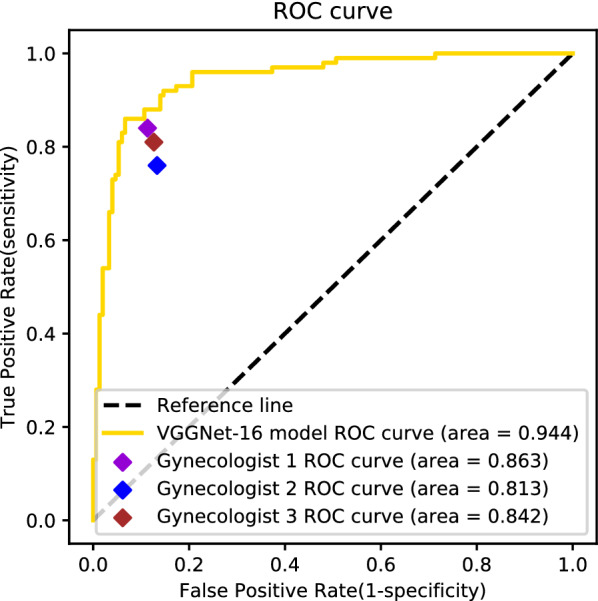
Binary ROC curves of the VGGNet-16 model and gynecologists. Binary receiver operating characteristic (ROC) curves for classifying lesions as premalignant/malignant or benign. The model curve is shown as a gold line and the curves for gynecologists 1, 2, and 3 are marked with purple, blue, and scarlet diamonds, respectively

When classifying premalignant/malignant and benign lesions, the accuracy, sensitivity, specificity, precision, f1-score, and AUC of the VGGNet-16 model were 90.8%, 83.0%, 96.0%, 93.3%, 87.8%, and 0.944. The accuracy of the three gynecologists was 86.8%, 82.4%, and 84.8%, and their AUCs were 0.863, 0.813, and 0.842, respectively. In this task, both the model and the gynecologists improved their performance significantly compared with the five-category classification task. Detailed two-category diagnostic performance evaluation metrics are shown in Table 3. The two-category ROC curve of the model and gynecologists is shown in Fig. 9.

**Table 3:** Diagnostic performance of the VGGNet-16 model and gynecologists in the two-category classification task

Classifier	Sensitivity (%)	Specificity (%)	Precision (%)	F1-score (%)	AUC	Accuracy (%)
VGGNet-16	83.0	96.0	93.3	87.8	0.944	90.8
Gynecologist 1	84.0	88.7	83.2	83.6	0.863	86.8
Gynecologist 2	76.0	86.7	79.2	77.6	0.813	82.4
Gynecologist 3	81.0	87.3	81.0	81.0	0.842	84.8

*AUC* area under the receiver operating characteristic (ROC) curve

### Comparison between model-aided diagnosis and direct diagnosis by gynecologists

After we split the test set equally at random, the test sets Part I and Part II were used for direct diagnosis and model-aided diagnosis by gynecologists. The accuracies of direct diagnosis of test set Part I by the three gynecologists were 64.0%, 62.4%, and 69.2%, respectively. Subsequently, gynecologists diagnosed the test set Part II with the aid of the model, and their accuracies were 78.4%, 72.8%, and 77.6%, respectively. The detailed comparison of the five-category diagnostic performance evaluation metrics is shown in Table 4. The five-category ROC curves of the gynecologists’ direct diagnoses and model-aided diagnoses are shown in Additional file 2: Figure S1. The confusion matrices of the direct diagnoses and model-aided diagnoses by gynecologists are shown in Additional file 3: Figure S2.

**Table 4:** Comparison of direct/model-aided diagnostic performance of the gynecologists in the five-category classification task

Category	Sensitivity (%)	Specificity (%)	Precision (%)	F1-score (%)	AUC	Accuracy (%)
Gynecologist 4						
EH	59.1	94.2	68.4	63.4	0.766	64.0
AH	58.6	93.8	73.9	65.4	0.762	
EC	56.5	89.2	54.2	55.3	0.729	
EP	78.3	85.3	54.5	64.3	0.818	
SM	67.9	92.8	73.1	70.4	0.803	
Model-aided gynecologist 4						
EH	82.1	93.8	79.3	80.7	0.880	78.4
AH	47.6	98.1	83.3	60.6	0.728	
EC	77.8	94.9	80.8	79.2	0.863	
EP	92.6	90.8	73.5	82.0	0.917	
SM	86.4	95.1	79.2	82.6	0.908	
Gynecologist 5						
EH	68.0	82.5	45.5	54.5	0.754	62.4
AH	55.2	93.8	72.7	62.7	0.745	
EC	52.2	90.2	54.5	53.3	0.712	
EP	73.9	88.2	58.6	65.4	0.811	
SM	64.3	99.0	94.7%	76.6	0.816	
Model-aided gynecologist 5						
EH	82.1	84.5	60.5	69.7	0.833	72.8
AH	42.9	92.3	52.9	47.4	0.676	
EC	74.1	96.9	87.0	80.0	0.855	
EP	81.5	94.9	81.5	81.5	0.882	
SM	77.3	97.1	85.0	81.0	0.872	
Gynecologist 6						
EH	59.1	96.1	76.5	66.7	0.776	69.2
AH	58.6	85.4	54.8	56.7	0.720	
EC	52.2	92.2	60.0	55.8	0.722	
EP	91.3	93.1	75.0	82.4	0.922	
SM	78.6	92.8	75.9	77.2	0.857	
Model-aided gynecologist 6						
EH	75.0	96.9	87.5	80.8	0.860	77.6
AH	57.1	94.2	66.7	61.5	0.757	
EC	77.8	93.9	77.8	77.8	0.858	
EP	88.9	89.8	70.6	78.7	0.893	
SM	86.4	97.1	86.4	86.4	0.917	

*AH* atypical hyperplasia, *AUC* area under the receiver operating characteristic (ROC) curve, *EC* endometrial cancer, *EH* endometrial hyperplasia without atypia, *EP* endometrial polyp, *SM* submucous myoma

## Discussion

This study explored the classification ability of the VGGNet-16 model for diagnosis of endometrial lesions using hysteroscopic images for the first time, with accuracies of 80.8% and 90.8% in our five-category and two-category classification tasks.

The benefit of CNN model is that the output provides the probability that a given hysteroscopy image belongs to each category. Even if the model makes a misclassification, the output contains a specific probability of the correct label. In contrast, it is difficult for hysteroscopists to give specific probabilities for their diagnoses. In most cases, gynecologists can only give two judgments: yes or no. The ability to harness probabilities is an important reason why the CNN model has a significantly higher AUC for each lesion type than the gynecologists. In this study, it has been confirmed that the model output probabilities can provide a convincing diagnostic reference for gynecologists and effectively reduce the subjectivity of gynecologists’ diagnoses. Although the CNN model is difficult to interpret [28], visualizing its calculations and outputs helps us to understand its working process.

In the absence of dynamic vision, diagnosis based only on static local hysteroscopy images led to lower sensitivity and specificity of the gynecologists’ diagnoses in this study as compared to results reported in a meta-analysis [29]. Given the appearance similarities of EH, AH, and EC endometrial lesions, it is relatively difficult for both the model and the gynecologists to distinguish between them. In actual clinical practice, hysteroscopists achieve better diagnostic performance through retrospective case data and dynamic vision. Gynecologists will give full consideration to the specific conditions of patients before performing hysteroscopy. For these difficult to distinguish endometrial lesions, gynecologists will actively advise patients to take pathological tissue specimens and submit them for examination during hysteroscopy to confirm the diagnosis and avoid over- or undertreatment. At this stage, the VGGNet-16 model in our study can only be used as an auxiliary diagnostic tool for gynecologists. Gynecologists can refer to the probability provided by the model and combine it with other clinical data to obtain a more accurate preliminary clinical diagnosis before the histopathological results are clear. In future research, we aim to implement a multimodal deep learning model that similarly combines case data and hysteroscopic images [30].

Machine learning and deep learning, an important branch of artificial intelligence, have also made outstanding contributions in the medical field, such as in clinical prediction models and radiomics [31, 32]. Regardless of the research direction, these artificial intelligence technologies have considerable clinical application value. We believe that each technology plays a different role in diagnosis, treatment, and the prediction of clinical outcomes. The integration of an artificial intelligence system into each medical subdiscipline, conforming to the clinical diagnosis and treatment process, is the ultimate goal.

The results of this study have demonstrated the feasibility of applying deep learning techniques to the diagnosis of endometrial lesions. Although there is a gap between the diagnostic performance of the model and the histopathological results in this study, under the experimental conditions of this study, the CNN model's ability to classify hysteroscopic images slightly exceeded that of the gynecologists and can provide gynecologists with objective references.

There are some limitations to our research. First, this study included only the five most common endometrial lesions, and lesions with low incidence were not included. Moreover, all images were collected from the same endoscopic camera system of the same hospital, thus the images may lack diversity. Finally, no prospective validation was performed in this study. We speculate that by expanding the dataset samples, the retrained model should achieve better diagnostic performance and generalization capability. Our group will collect more data at multiple centers to retrain the model and implement prospective validation. The model that obtains better diagnostic performance will be considered for application to clinical practice.

## Conclusions

In this study, we developed the first CNN-based CAD system for diagnostic hysteroscopy image classification. The VGGNet-16 model used in our study shows comparable diagnostic performance to expert gynecologists in classifying five types of endometrial lesion images. The model can provide objective diagnostic evidence for hysteroscopists and has potential clinical application value.

## Supplementary Information

**Additional file 1: Table S1.** Summary of fine-tuned VGGNet-16 model. Conv3: 3 × 3 convolutional layer; ReLU: rectified linear unit.

**Additional file 2: Figure S1.** Five-category ROC curves of the gynecologists’ direct diagnoses and model-aided diagnoses. Five-category receiver operating characteristic (ROC) curves: a, c, and e are the direct diagnostic ROC curves of gynecologists 4, 5, and 6, respectively. b, d, and f are the model-aided diagnostic ROC curves of gynecologists 4, 5, and 6, respectively. AH: atypical hyperplasia; EC: endometrial cancer; EH: endometrial hyperplasia without atypia; EP: endometrial polyp; SM: submucous myoma.

**Additional file 3: Figure S2.** Confusion matrices of the gynecologists’ direct diagnoses and model-aided diagnoses. Confusion matrices: a, c, and e are the direct diagnostic confusion matrices of gynecologists 4, 5, and 6, respectively. b, d, and f are the model-aided diagnostic confusion matrices of gynecologists 4, 5, and 6, respectively. The x axes are the predicted labels, which are the diagnoses made by the gynecologists or model-aided gynecologists. The y axes are the true labels, which is the histopathological result. The number in each small square represents the corresponding number of images with the same predicted true label and its percentage of the total number of images under the true label. AH: atypical hyperplasia; EC: endometrial cancer; EH: endometrial hyperplasia without atypia; EP: endometrial polyp; SM: submucous myoma.

## Data Availability

All data presented in this study are included in the article/additional material.

## Abbreviations

AH: Atypical hyperplasia
AUC: Area under the receiver operating characteristic curve
CAD: Computer-aided diagnosis
CNN: Convolutional neural network
EC: Endometrial cancer
EH: Endometrial hyperplasia without atypia
EP: Endometrial polyp
FCNN: Fully convolutional neural network
FN: False negative
FP: False positive
ILSVRC: ImageNet Large Scale Visual Recognition Challenge
ROC: Receiver operating characteristic
SM: Submucous myoma
SGD: Stochastic gradient descent
TN: True negative
TP: True positive
t-SNE: T-distributed stochastic neighbor embedding
