# Supplementary material for: Deep learning model for classifying endometrial lesions
Source: J Transl Med. 2021 Jan 6;19:10. doi: 10.1186/s12967-020-02660-x (PMC7788977; doi:10.1186/s12967-020-02660-x)
Supplement: Supplementary file 1 — Additional file 1: Table S1. Summary of fine-tuned VGGNet-16 model. Conv3: 3 × 3 convolutional layer; ReLU: rectified linear unit. [file 12967_2020_2660_MOESM1_ESM.docx]

**Table S1. Summary of fine-tuned VGGNet-16 model**

| **Block** | **Layer** | **Output shape** | **Parameters** | **Activation** |
| --- | --- | --- | --- | --- |
| - | Input | (None,224,224,3) | 0 | - |
| Block1 | Conv3 | (None,224,224,64) | 1792 | ReLU |
| Block1 | Batch normalization | (None,224,224,64) | 256 | - |
| Block1 | Conv3 | (None,224,224,64) | 36928 | ReLU |
| Block1 | Batch normalization | (None,224,224,64) | 256 | - |
| Block1 | MaxPool | (None,112,112,64) | 0 | - |
| Block2 | Conv3 | (None,112,112,128) | 73856 | ReLU |
| Block2 | Batch normalization | (None,112,112,128) | 512 | - |
| Block2 | Conv3 | (None,112,112,128) | 147584 | ReLU |
| Block2 | Batch normalization | (None,112,112,128) | 512 | - |
| Block2 | MaxPool | (None,56,56,128) | 0 | - |
| Block3 | Conv3 | (None,56,56,256) | 295168 | ReLU |
| Block3 | Batch normalization | (None,56,56,256) | 1024 | - |
| Block3 | Conv3 | (None,56,56,256) | 590080 | ReLU |
| Block3 | Batch normalization | (None,56,56,256) | 1024 | - |
| Block3 | Conv3 | (None,56,56,256) | 590080 | ReLU |
| Block3 | Batch normalization | (None,56,56,256) | 1024 | - |
| Block3 | MaxPool | (None,28,28,256) | 0 | - |
| Block4 | Conv3 | (None,28,28,512) | 1180160 | ReLU |
| Block3 | Batch normalization | (None,28,28,512) | 2048 | - |
| Block4 | Conv3 | (None,28,28,512) | 2359808 | ReLU |
| Block3 | Batch normalization | (None,28,28,512) | 2048 | - |
| Block4 | Conv3 | (None,28,28,512) | 2359808 | ReLU |
| Block3 | Batch normalization | (None,28,28,512) | 2048 | - |
| Block4 | MaxPool | (None,14,14,512) | 0 | - |
| Block5 | Conv3 | (None,14,14,512) | 2359808 | ReLU |
| Block5 | Batch normalization | (None,14,14,512) | 2048 | - |
| Block5 | Conv3 | (None,14,14,512) | 2359808 | ReLU |
| Block5 | Batch normalization | (None,14,14,512) | 2048 | - |
| Block5 | Conv3 | (None,14,14,512) | 2359808 | ReLU |
| Block5 | Batch normalization | (None,14,14,512) | 2048 | - |
| Block5 | MaxPool | (None,7,7,512) | 0 | - |
| - | Flatten | (None,25088) | 0 | - |
| - | Fully connected | (None,512) | 12845568 | ReLU |
| - | Dropout | (None,512) | 0 | - |
| - | Fully connected | (None,512) | 262656 | ReLU |
| - | Dropout | (None,512) | 0 | - |
| - | Fully connected | (None,5) | 2565 | Softmax |

Conv3: 3 × 3 convolutional layer; ReLU: rectified linear unit
