# Supplementary figures and images for: Deep learning model for classifying endometrial lesions
Source: J Transl Med. 2021 Jan 6;19:10. doi: 10.1186/s12967-020-02660-x (PMC7788977; doi:10.1186/s12967-020-02660-x)

**a**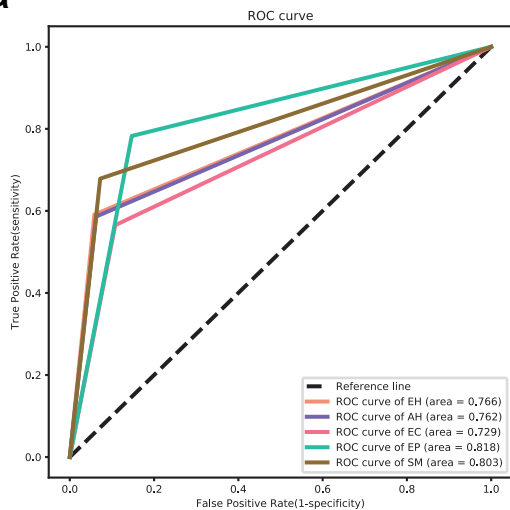**b**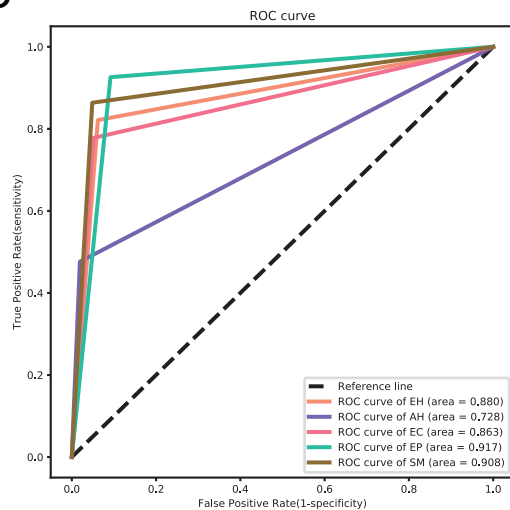**c**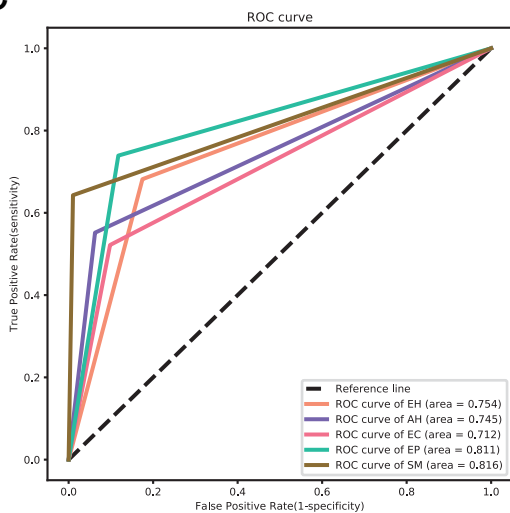**d**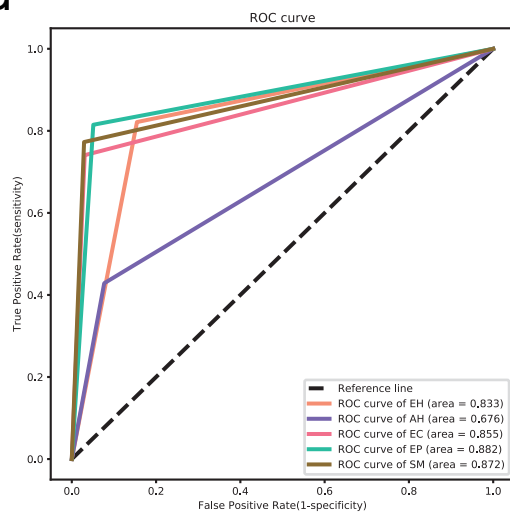**e**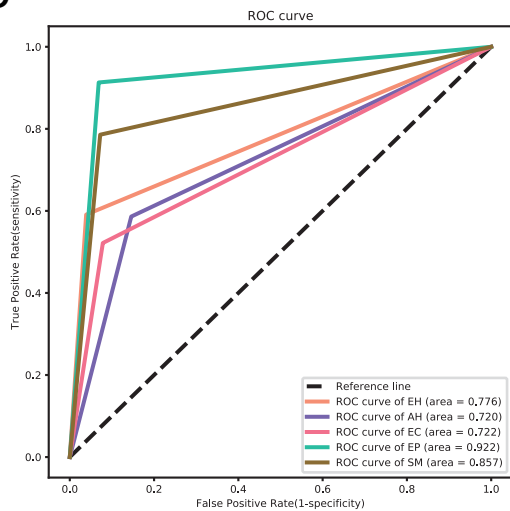**f**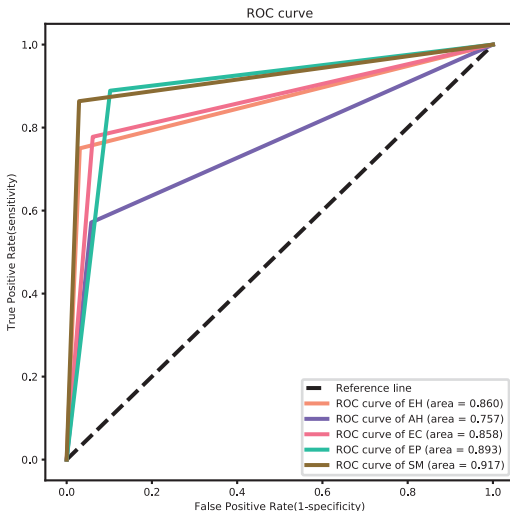

Supplement: Supplementary file 2 — Additional file 2: Figure S1. Five-category ROC curves of the gynecologists’ direct diagnoses and model-aided diagnoses. Five-category receiver operating characteristic (ROC) curves: a, c, and e are the direct diagnostic ROC curves of gynecologists 4, 5, and 6, respectively. b, d, and f are the model-aided diagnostic ROC curves of gynecologists 4, 5, and 6, respectively. AH: atypical hyperplasia; EC: endometrial cancer; EH: endometrial hyperplasia without atypia; EP: endometrial polyp; SM: submucous myoma. [file 12967_2020_2660_MOESM2_ESM.pdf]
