# Supplementary material for: Deep learning model for classifying endometrial lesions
Source: J Transl Med. 2021 Jan 6;19:10. doi: 10.1186/s12967-020-02660-x (PMC7788977; doi:10.1186/s12967-020-02660-x)
Supplement: Supplementary file 3 — Additional file 3: Figure S2. Confusion matrices of the gynecologists’ direct diagnoses and model-aided diagnoses. Confusion matrices: a, c, and e are the direct diagnostic confusion matrices of gynecologists 4, 5, and 6, respectively. b, d, and f are the model-aided diagnostic confusion matrices of gynecologists 4, 5, and 6, respectively. The x axes are the predicted labels, which are the diagnoses made by the gynecologists or model-aided gynecologists. The y axes are the true labels, which is the histopathological result. The number in each small square represents the corresponding number of images with the same predicted true label and its percentage of the total number of images under the true label. AH: atypical hyperplasia; EC: endometrial cancer; EH: endometrial hyperplasia without atypia; EP: endometrial polyp; SM: submucous myoma. [file 12967_2020_2660_MOESM3_ESM.pdf]

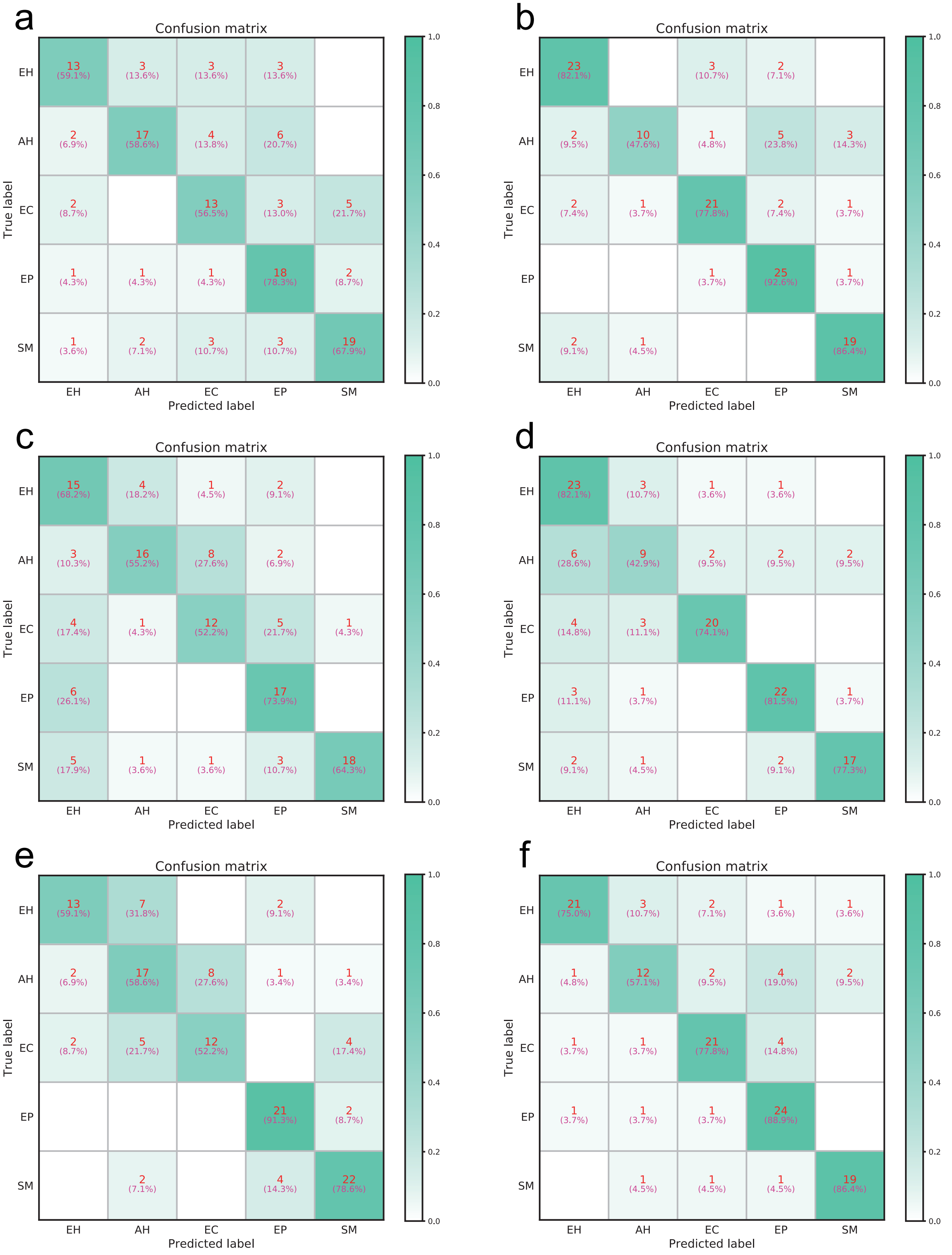
a
Confusion matrix
True label
Predicted label
EH
AH
EC
EP
SM
13 (59.1%) 3 (13.6%) 3 (13.6%) 3 (13.6%)
2 (6.9%) 17 (58.6%) 4 (13.8%) 6 (20.7%)
2 (8.7%) 13 (56.5%) 3 (13.0%) 5 (21.7%)
1 (4.3%) 1 (4.3%) 1 (4.3%) 18 (78.3%) 2 (8.7%)
1 (3.6%) 2 (7.1%) 3 (10.7%) 3 (10.7%) 19 (67.9%)
0.0
0.2
0.4
0.6
0.8
1.0
b
Confusion matrix
23 (82.1%) 3 (10.7%) 2 (7.1%)
2 (9.5%) 10 (47.6%) 1 (4.8%) 5 (23.8%) 3 (14.3%)
2 (7.4%) 1 (3.7%) 21 (77.8%) 2 (7.4%) 1 (3.7%)
1 (3.7%) 25 (92.6%) 1 (3.7%)
2 (9.1%) 1 (4.5%) 19 (86.4%)
c
Confusion matrix
15 (68.2%) 4 (18.2%) 1 (4.5%) 2 (9.1%)
3 (10.3%) 16 (55.2%) 8 (27.6%) 2 (6.9%)
4 (17.4%) 1 (4.3%) 12 (52.2%) 5 (21.7%) 1 (4.3%)
6 (26.1%) 17 (73.9%)
5 (17.9%) 1 (3.6%) 1 (3.6%) 3 (10.7%) 18 (64.3%)
d
Confusion matrix
23 (82.1%) 3 (10.7%) 1 (3.6%) 1 (3.6%)
6 (28.6%) 9 (42.9%) 2 (9.5%) 2 (9.5%) 2 (9.5%)
4 (14.8%) 3 (11.1%) 20 (74.1%)
3 (11.1%) 1 (3.7%) 22 (81.5%) 1 (3.7%)
2 (9.1%) 1 (4.5%) 2 (9.1%) 17 (77.3%)
e
Confusion matrix
13 (59.1%) 7 (31.8%) 2 (9.1%)
2 (6.9%) 17 (58.6%) 8 (27.6%) 1 (3.4%) 1 (3.4%)
2 (8.7%) 5 (21.7%) 12 (52.2%) 4 (17.4%)
21 (91.3%) 2 (8.7%)
2 (7.1%) 4 (14.3%) 22 (78.6%)
f
Confusion matrix
21 (75.0%) 3 (10.7%) 2 (7.1%) 1 (3.6%) 1 (3.6%)
1 (4.8%) 12 (57.1%) 2 (9.5%) 4 (19.0%) 2 (9.5%)
1 (3.7%) 1 (3.7%) 21 (77.8%) 4 (14.8%)
1 (3.7%) 1 (3.7%) 1 (3.7%) 24 (88.9%)
1 (4.5%) 1 (4.5%) 1 (4.5%) 19 (86.4%)
